# High resolution genomic analysis of sporadic breast cancer using array-based comparative genomic hybridization

**DOI:** 10.1186/bcr1356

**Published:** 2005-11-24

**Authors:** Tara L Naylor, Joel Greshock, Yan Wang, Theresa Colligon, QC Yu, Virginia Clemmer, Tal Z Zaks, Barbara L Weber

**Affiliations:** 1 Abramson Family Cancer Research Institute, University of Pennsylvania School of Medicine, Philadelphia, PA 19104, USA; 2 St Francis Hospital, Wilmington, DE 19805, USA; 3 GlaxoSmithKline, King of Prussia, PA 19406, USA

## Abstract

**Introduction:**

Genomic aberrations in the form of subchromosomal DNA copy number changes are a hallmark of epithelial cancers, including breast cancer. The goal of the present study was to analyze such aberrations in breast cancer at high resolution.

**Methods:**

We employed high-resolution array comparative genomic hybridization with 4,134 bacterial artificial chromosomes that cover the genome at 0.9 megabase resolution to analyze 47 primary breast tumors and 18 breast cancer cell lines.

**Results:**

Common amplicons included 8q24.3 (amplified in 79% of tumors, with 5/47 exhibiting high level amplification), 1q32.1 and 16p13.3 (amplified in 66% and 57% of tumors, respectively). Moreover, we found several positive correlations between specific amplicons from different chromosomes, suggesting the existence of cooperating genetic loci. Queried by gene, the most frequently amplified kinase was *PTK2 *(79% of tumors), whereas the most frequently lost kinase was *PTK2B *(hemizygous loss in 34% of tumors). Amplification of *ERBB2 *as measured by comparative genomic hybridization (CGH) correlated closely with *ERBB2 *DNA and RNA levels measured by quantitative PCR as well as with ERBB2 protein levels. The overall frequency of recurrent losses was lower, with no region lost in more than 50% of tumors; the most frequently lost tumor suppressor gene was *RB1 *(hemizygous loss in 26% of tumors). Finally, we find that specific copy number changes in cell lines closely mimicked those in primary tumors, with an overall Pearson correlation coefficient of 0.843 for gains and 0.734 for losses.

**Conclusion:**

High resolution CGH analysis of breast cancer reveals several regions where DNA copy number is commonly gained or lost, that non-random correlations between specific amplicons exist, and that specific genetic alterations are maintained in breast cancer cell lines despite repeat passage in tissue culture. These observations suggest that genes within these regions are critical to the malignant phenotype and may thus serve as future therapeutic targets.

## Introduction

Genomic instability is a hallmark of cancer, and specific subchromosomal copy number changes are thought to play a driving role in the transformation of normal cells to malignant clones. These genomic copy number changes may result in deletion of one or both alleles of tumor suppressor genes, overexpression of oncogenes and rearrangements that may alter transcription of target and downstream genes (reviewed in [1]). Several recent studies suggest that fixed genetic abnormalities in human cancers may be highly predictive of response to targeted therapeutics. For example, *ERBB2 *amplification may be more predictive of response to trastuzumab than protein overexpression with normal gene copy number (reviewed in [2]), and activating mutations in *EGFR *determine response to gefitinib [3,4].

There is an extensive literature on DNA copy number alterations in cancer using low resolution technology such as PCR-based allelotyping, spectral karyotyping, and metaphase comparative genomic hybridization (CGH). These studies, however, are limited in their ability to characterize specific abnormalities across the genome and to identify altered genes within the large regions defined by these methodologies. Nonetheless, when considering the breast cancer literature, these studies are consistent, frequently reporting the same regions of gain (1q, 8q, 11q, 17q, 20q) and loss (6q, 8p, 9p, 13q, 16q) [5-11].

More recent studies have employed higher resolution array-based CGH (aCGH) to characterize primary tumors [10,12-14]. These studies demonstrate the enormous complexity of cancer genomes, but also provide evidence that consistent, non-random patterns of copy number alterations are present in human cancers and support the hypothesis that selection for genomic changes conferring a proliferative advantage plays an important role in malignant transformation.

To further characterize the genomic alterations that may drive both transformation and response to targeted therapies, we developed an aCGH platform that covers the genome at 0.9 megabase (Mb) resolution [15]. Here we report the use of these arrays to define the genomic profile of 47 primary breast tumors and 18 breast cancer cell lines. Specifically, we evaluated the most common regions of gain and loss across the genome, assessed correlations with clinical parameters, characterized the *ERRB2 *locus and pathway in detail, and identified potentially cooperating genetic loci.

## Materials and methods

### Cell lines and tumor samples

Eighteen breast cancer cell lines (BT-20, HCC1143, HCC1187, HCC1395, HCC1419, HCC1569, HCC1599, HCC1937, HCC1954, HCC202, HCC2218, HCC38, MDA-MB-134-VI, MDA-MB-157, MDA-MB-361, MDA-MB-415, SKBR-3, and T-47D) were obtained from American Type Culture Collection (Manassas, VA, USA). Forty-seven fresh-frozen primary breast tumors (thirty-nine infiltrating ductal carcinoma (IDC), two infiltrating lobular carcinoma (ILC), four mixed IDC/ILC, two ductal carcinoma *in situ*) were obtained from St Francis Hospital (Wilmington, DE, USA). Tissue and data collection were performed with patient consent as approved by the Institutional Review Boards of both The University of Pennsylvania and St Francis Hospital institutions. Tumors not required for diagnosis were frozen in liquid nitrogen and used for further study. Clinical data and tumor characteristics are provided in Additional file 1.

### DNA copy number detection

Hematoxylin and eosin staining was used to define a region of tissue containing at least 70% tumor, which was dissected away from normal tissue using a scalpel. Thirty 20 μm sections were used for DNA isolation by proteinase K digestion followed by phenol/chloroform extraction in PhaseLock Gel tubes (Eppendorf, Westbury, NY, USA). An additional hematoxylin and eosin stained section was used to verify the persistence of at least 70% tumor in the residual tissue adjacent to the sectioned area. Normal genomic DNA, used as the reference probe for aCGH co-hybridization, was prepared from peripheral leukocytes of healthy female volunteers of diverse ethnic backgrounds using alkaline lysis. DNA from at least five donors was pooled equally before labeling.

CGH arrays were prepared using degenerate oligonucleotide-primed PCR products from 4,134 bacterial artificial chromosome (BAC) clones printed in triplicate on glass slides (Ultra GAPS, Corning, Acton, MA, USA) [15].

For hybridization, 1 μg of test DNA and 1 μg of pooled normal human DNA were labeled with either Cy3-dCTP or Cy5-dCTP incorporated by random priming (Bioprime Labeling Kit, Invitrogen, Carlsbad, CA, USA). After overnight incubation at 37°C, labeling reactions were purified (MinElute PCR Clean-up, Qiagen, Valencia, CA, USA), and the tumor and normal DNAs combined and ethanol-precipitated with 100 μg human Cot-1 DNA (Invitrogen). DNAs were rehydrated in 50 μl of formamide-based hybridization buffer [16], denatured at 70°C for 15 minutes and re-annealed for 30 minutes at 37°C to block repetitive sequences. Tumor and normal DNAs were then co-hybridized to the CGH arrays at 37°C for 72 h on a rotating platform and washed as described by Gray and colleagues [16]. For each sample, the test and reference DNA also were labeled with the opposite dye in a separate experiment ('dye swap') to account for differences in dye incorporation and provide additional data points for analysis.

Arrays were scanned on a GenePix 4000B scanner (Axon Instruments, Downingtown, PA, USA) and the composite tiff image was segmented using GenePix Pro 4.0 (Axon Instruments). Foreground (signal) and background intensities were generated separately for Cy3 and Cy5 channels, and the local background intensity was subtracted to generate a corrected intensity for each spot. The ratio of background corrected Cy3 to Cy5 values was then calculated for each spot and, because each BAC clone was printed on the array three times, these measurements were averaged to generate the intensity ratio (IR) for the clone. Two arrays (dye swap) were hybridized for each sample; therefore, a total of six measurements determine the relative DNA copy number of each BAC clone in the test sample relative to the reference sample.

Data were normalized and visualized using CGHAnalyzer [15], which is available for download from CGHcloneDB [17]. Copy number deviations from diploid were determined by BACs with IRs that differed significantly from a normal distribution representing diploid copy number which was developed using 50 normal:normal genomic DNA hybridizations on these arrays [15] (>2 standard deviations for both replicates). We use the following thresholds and terms throughout the text: IR >2.0 (high-level copy number gain, >5 copies), IR <0.5 (homozygous deletion).

### DNA and transcript copy number validation

Genomic copy number alterations of selected regions were validated using a relative quantitative (Q)-PCR assay. For the *ERBB2 *region, the LightCycler HER2/*neu *DNA Quantification Kit (Roche, Nutley, NJ, USA) was used. An additional 50 × 20 μm sections were cut from 18 primary tumors (15 IDC, one mixed IDC/ILC, two ductal carcinoma *in situ*) directly into Trizol for RNA extraction and the RNA was further purified using the RNeasy Kit (Qiagen). *ERBB2 *transcript levels were determined using the High Capacity cDNA Archive Kit, and a TaqMan^© ^Microfluidic Card Assay on Demand (Hs. 00170433_m1; Applied Biosystems, Foster City, CA, USA). The 18S ribosomal subunit assay (Hs. 99999904_m1) was used as a control.

DNA copy number for PTK2 (chromosome 8, 141,639,559 to 141,781,701 bases) was determined by real-time Q-PCR using TaqMan Universal PCR master mix and TaqMan primers/probe designed using Primer Express software (Applied Biosystems). The data were collected using the Applied Biosystems Prism 7900HT Sequence Detection System, analyzed with SDS v2.1 and Excel (Microsoft Corp., Redmond, WA, USA). PTK2 levels are reported relative to TBP (TATA box binding protein), which is diploid in the breast tumors. Primers used were: PTK2-QF, 5' TGACTATTTTACAGCCACTGGAGTTAA3'; PTK2-QR, 5' GAAAACCAAATTCCTGTTTTGCTT 3'; PTK2-QP: 5'FAM ACCCTTCCTTGTATCTGTCTTCCCAGGAGA TAMRA 3'.

These data were directly compared to aCGH data from a BAC clone covering the *PTK2 *locus (RP11-502G13; chromosome 8, 142,176,518 to 142,176,951 bases) and several neighboring BACS. The concordance of these data was assessed by a t-test between the Taqman^© ^data for those samples with and without high-level gains (BAC clone IR >2.0).

### Statistical analysis

As only a portion of the genome is directly covered by BAC clones on this array, a flanking region algorithm built into CGHAnalyzer extrapolates copy number estimations in uncovered regions between BAC clones. This extrapolates the extent of the copy number alteration represented by a given BAC to the genomic coordinate of the neighboring BAC clone of a different estimated copy number. This approach avoids missing important genetic changes between BACs but, by definition, overestimates the size of alterations. Consecutive BAC clones that are designated as being similarly altered are merged into a single representative region of change. The resultant data structure for a single sample is simply a series of genomic regions designated as either gained or lost. Pearson correlation coefficients were used to evaluate similarities (positive correlations) and differences (negative correlations) in copy number alteration trends within and across data sets (e.g., cell lines versus tumors). Pearson correlations were calculated by weighting the alteration frequency of each locus on a linear scale. Further, all correlations were compared to the distribution of correlations where the copy number alterations were arranged randomly (n = 1,000). To estimate the total portion of the genome gained or lost, each segment was summed and divided by 2,679 Mb, the total Mb in the genome (excluding heterochromatic, centromeric and telomeric regions not covered by BACs, and the sex chromosomes). The Wilcoxon rank sum test was used to compare estimates between tumors and cell lines, as well as between tumor subsets.

Correlation matrices were generated to identify the intersection of changes at two loci by a binomial probability-based metric. Specifically, the relative correlation of two loci was scored by their pair-wise comparison based on the genomic regions data structure, and defined in terms of the probability of the number of samples sharing aberrations at two loci if they were distributed among the samples by chance. The Fisher's exact test was used with permutation analysis to determine the statistical significance of the correlation between the most frequently aberrant loci in the data set. Multiple iterations (n = 2,000) were performed and the lowest p-values from these randomized iterations were compared to the p-values from the experimental data. Loci were considered significantly correlated when the associated p-value was less than the lowest p-value from the randomized data.

## Results

As the initial step in our analysis, we reviewed the existing literature on whole genome DNA copy number analysis of human breast cancers; four chromosomal CGH studies [5-8] and two array-based CGH studies [9,10] were used for comparison (Table 1). All studies identified recurrent gains on chromosomes 1q, 8q, 11q, 17q, 20q, and losses on 6q, 8p, 9p, 13q, 16q. However, our high-resolution arrays detected a higher percentage of tumors with these gains, as well as several high-level amplifications (IR >2, estimated >5 copies), in these regions. For example, six previous studies [5-10] suggest that 40% to 50% of primary breast tumors have copy number gains of chromosome 8q24; however, this region was amplified in 79% of the tumors in the current study. Our aCGH arrays also identified five regions of gain in more than 50%, and four regions of loss in more than 30% of tumors that have not been previously associated with breast cancer (Table 1).

### Comparison of primary tumors and cell lines

**Figure 1:**
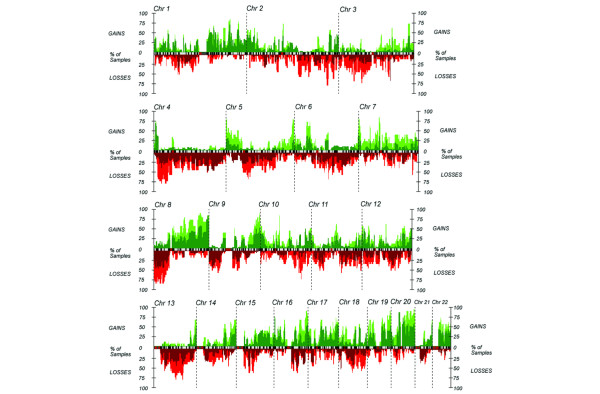
Array-based comparative genomic hybridization frequency plots of breast tumors and cell lines. Plots of primary breast tumors (light) overlaid onto breast cell lines (dark) with gains in green and losses in red.

We compared the location, frequency and size of copy number changes in primary tumors versus cell lines. Surprisingly, the location of more frequent gains and losses in the cell lines very closely mirrored those in the primary tumors (Fig. 1). This is demonstrated by a Pearson correlation coefficient of 0.843 for gains and 0.734 for losses. The mean correlation of randomly placed gains was 0.295 (σ = 0.078) and losses was 0.203 (σ = 0.082), which yield p < 0.0001 for the tumor versus cell line correlations for both cases when modeled to a normal distribution. Additionally, there appeared to be more alterations in cell lines. Primary tumors had gains involving a mean of 410.8 Mb (14.5%) of the genome, which was significantly greater than that seen in cell lines (μ = 674.2 Mb (23.8%), σ = 243.6 Mb (8.6%); p = 0.0014). Similarly, losses in primary tumors (μ = 286.1 Mb (10.1%), σ = 195.5 Mb (6.9%)) were less extensive than those seen in cell lines (μ = 589.3 Mb (20.8%), σ = 226.6 Mb (8.0%); p = 0.0001). As expected, the overall aberration rate was lower for primary tumors (μ = 696.9 Mb (24.6%), σ = 255.0 Mb (14.5%)) than cell lines (μ = 1269.2 Mb (44.8%), σ = 433.4 Mb (15.3%); p < 0.0001).

### Frequent amplifications

We identified 55 regions of gain present in more than 30% of the primary tumors (Additional file 2), ranging in size from 0.1 to 8.7 Mb (median 2.1 Mb). Of those 55 regions, 20 encompassed a region of estimated high-level copy number change with an IR >2 (range 2.1 to 8.7) in more than one tumor (Table 2). Several of these regions contain genes known to be amplified in breast cancer, including *ERBB2*, *EGFR *and *MYC*, while others include genes not previously implicated in breast cancer, including *PTK2*.

**Figure 2:**
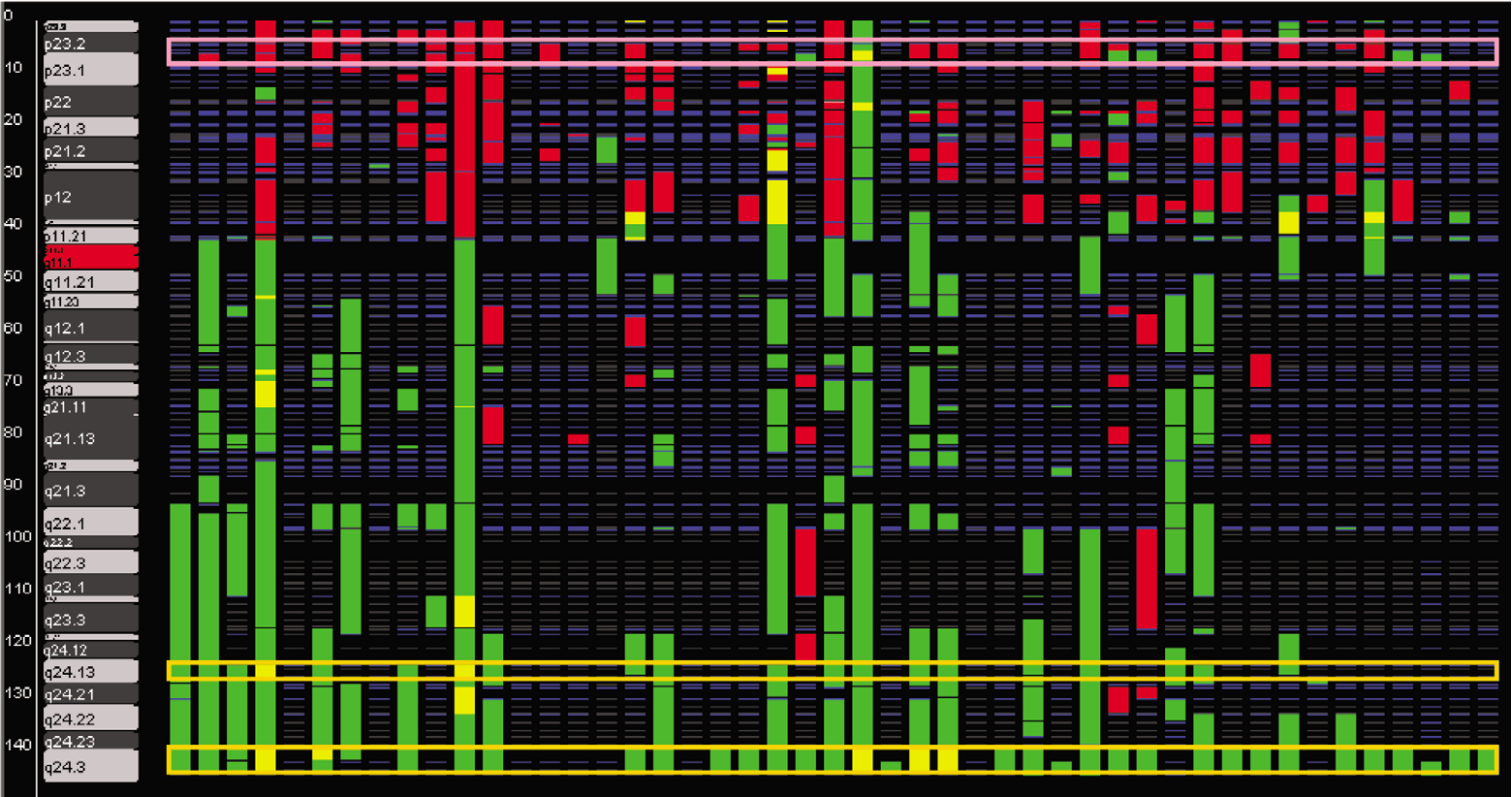
Comparative genomic hybridization analyzer view of chromosome 8. Tumors are displayed vertically and bacterial artificial chromosomes (BACS) by genome position horizontally. Low-level gains (ratio between 1.2 and 2.0) are green, high-level amplifications (ratio >2.0) are yellow, and single copy losses are red. Boxes surround common areas of aberration listed in Table 1. All BAC clone, gene and cytoband locations are given in megabase coordinates based on the UCSC genome browser (July 2003 freeze) [35].

The most frequently gained region in this sample set is chromosome 8q24. Metaphase CGH suggests that this region is a single amplicon, but with the increased resolution of aCGH, two distinct regions of gain become apparent (Fig. 2). The centromeric amplicon extends from 117.8 to 125.8 Mb (8q24.11-24.13), has a minimal common region of overlap (CRO) of 8.7 Mb, and is present in 20/47 tumors (43%) and 14/18 cell lines (78%). This CRO contains *MYC *and 23 other genes. Two of the 20 primary tumors and 3/18 cell lines with gains in this region have estimated high-level gains. The telomeric 8q24 amplicon extends from 139.3 to 144.8 Mb (8q24.3), and has a minimal CRO of 5.6 Mb. This is the most commonly gained region in the sample set, found in 37/47 primary tumors (79%) and 15/18 cell lines (83%). In this region, 5/47 primary tumors and 10/18 cell lines have high level copy number gains. This region includes *PTK2 *(*FAK*) as well as *GPR20*, *BAI1*, *ARC*, *JRK*, *PSCA*, *ARS*, *LYNX1*, *LY6D*, *GML*, *CYP11B1*, *CYP11B2*, *LY6E*, *HHCM*, *LY6H*, *TOP1MT*, *RHPN1*, *COL22A1*, *KCNK9*, *CHRAC1*, and *EIF2C2*.

Two other regions of copy number gains were detected in more than 50% of primary tumors. These regions are chromosome 1q32.1 (202.1 to 202.9 Mb, CRO 0.8 Mb), found in 31/47 of primary tumors (66%), and chromosome 16p13.3 (3.2 to 3.3 Mb, CRO 0.1 Mb) found in 27/47 primary tumors (57%). These gains were found in 14/18 (78%) and 11/18 cell lines (61%), respectively. The region on 1q32.1 contains *CNTN2*, *RBBP5*, *DustyPK*, *HUCEP11*, *SNARK*, *PCTK3*, *ELK4*, *Prostein*, and *NUCKS*. The region on 16p13.3 contains *ZNF205*, *ZNF215 and ZNF200*. The region on 17q12 containing *ERBB2 *(33.6 to 38.9 Mb, CRO 5.3 Mb) was also a commonly gained region in the primary tumors, with increased copy number detected in 21/47 primary tumors (45%) and 12/18 cell lines (67%). Three primary tumors and two cell lines had high-level gains.

### Frequent deletions

The frequency of recurrent losses was lower than that of gains; no region was lost in more than 50% of primary tumors (Table 1). There were 13 regions of hemizygous loss found in at least 30% of tumors. These regions had CROs ranging in size from 0.1 to 4.0 Mb (median 1.3 Mb).

The two most frequently deleted regions occurred in 40% (19/47) of the primary tumors. The first, 8p23.1-23.2 (4.8 to 7.6 Mb, CRO 2.8 Mb), contains *LPAAT-e*, *SPAG11 *and many members of the defensin family. This region was deleted in 15/18 cell lines (83%). Of note, three of these cell lines had an IR <0.5, which is indicative of a homozygous deletion. The second, 4q31.1-31.21 (141.6 to 145.2 Mb, CRO 3.6 Mb), contains a region not previously described as frequently deleted in breast cancer. This region was deleted in 6/18 cell lines (33%), and contains six known genes: *SCOC*, *CLGN*, *UCP1*, *ZNF330*, *IL15*, and *INPP4B*.

The only putative homozygous deletion in a primary tumor (IR <0.5 within a region of hemizygous loss) was on 9p21.2 (27.6 to 27.9 Mb) and was seen in 2/47 primary tumors and 2/18 cell lines. This region contains *ELAV*, *PLAA*, *CCDC2*, *LRRC19*, *TEK*, *MOB3B*, and *IFNK*. In contrast, we identified 57 putative homozygous deletions in cell lines. There were several recurring homozygous deletions; two were found in three cell lines and eight were found in two cell lines (Table 3). The size of these regions ranged from 0.1 to 6.0 Mb (median 3.2 Mb). All of the recurrent homozygous deletions occurred in regions of hemizygous loss detected in >10% of primary tumors (median 23.5%; range 13% to 40%). Three of the putative recurrent homozygous deletions contain a known cancer-related gene; 8p22-21.3 (17.9 to 22.0 Mb; PCM1), 8p21.2 (23.0 to 27.1 Mb; TNFRS10A) and 18q21.1-21.2 (46.8 to 52.8 Mb; MADH4) (cancer gene list queried from [15]). None of these regions contain known fragile sites.

### Correlation of genomic alterations with clinical characteristics

Based on the flanking region approach to copy number alteration estimation, the mean percent of the genome gained and lost was calculated for each of the tumor subgroups (e.g., estrogen receptor positive versus negative) and differences evaluated using the Wilcoxon rank sum test (Additional file 3). In this sample set, total percentage of the genome altered did not vary significantly by stage (p = 0.79), grade (p = 0.12), *ERBB2 *status (p = 0.48), ER status (p = 0.23), menopausal status (p = 0.89), or DNA ploidy (p = 0.70). Similar results were observed when individually evaluating the percentage of the genome gained or lost. No obvious correlations between specific gains or losses were observed, possibly as a result of small numbers of patients in each subgroup in relation to a large number of aberrations.

### Genes with frequent copy number changes

We queried the frequency of copy number changes in all known genes, as well as the following gene classes; tumor suppressor genes [18], kinases [19], and cancer-related genes adapted from work by Futreal and colleagues [15,20]. The 10 most frequent gains and losses for each gene class are shown in Additional file 4 (complete list available on request). *RB1 *was the most frequently lost tumor suppressor gene, hemizygously deleted in 12/47 primary tumors (26%) and 9/18 cell lines (50%). *RB1 *copy number gains were not seen in any primary tumors and only 1/18 cell lines (5.6%).

The ten most frequently amplified kinases were gained at least six times more frequently than lost, likely indicating a selection for gains of these genes. *PTK2 *was the most frequently gained gene on both the kinase and cancer-related gene list, amplified in 37/47 primary tumors (79%) and 15/18 cell lines (83%). *PTK2 *was never deleted in the primary tumors and deleted in only 2/18 cell lines (11%). Amplification of the *PTK2 *gene within the amplified locus was further analyzed by Taqman quantification, and DNA levels in samples that had amplified *PTK2 *(IR >2.0 by aCGH) were significantly higher than levels in unamplified samples (p = 0.0018; data not shown). Interestingly, five kinases (*PTK2B*, *PHKB*, *DCAMKL1*, *TEK*, *MAP2K4*) were deleted at least five times more frequently than gained, suggesting these kinases may play a role in negatively regulating growth. Of note, inactivating mutations in *MAP2K4 *have been identified in 5% of breast cancers [19]. Additionally, *PTK2B *is both the most frequently lost kinase and cancer-related gene, hemizygously deleted in 16/47 tumors (34%), and 12/18 cell lines (67%). The remaining five of the ten most frequently deleted kinases are found with equal frequency in regions of gain and loss, an indication that copy number changes in these genes are not likely to be functionally significant and consistent with the idea that most kinases confer a proliferative advantage.

Tumor suppressor genes and kinases are subsets of the cancer-related gene list, so it is not surprising that seven of the top ten cancer-related gene gains are kinases. However, there are three cancer-related genes (*GRB2*, *GAS6*, *MLLT6*) found in regions of gain at least five times more frequently than lost that are not kinases. *GRB2*, an adaptor molecule in the epidermal growth factor (EGF) signaling pathway, is gained in 22/47 primary tumors (47%) and 12/47 cell lines (67%). *GAS6*, the ligand of the tyrosine kinase AXL, is gained in 21/47 primary tumors (45%) and 12/47 cell lines (67%). Two cancer-related genes (*RBL2 *and *CDH8*) are three times more frequently lost than gained. *RBL2/p130 *is deleted in 14/47 primary tumors (30%) and 10/18 cell lines (56%).

### Correlation of *ERBB2 *DNA, RNA and protein levels

We identified five distinct regions of gain on chr17q, including the *ERBB2 *locus at 17q12. Because of the clinical significance of this gene, we determined *ERBB2 *genomic DNA copy number (Q-PCR LightCycler) and mRNA transcript levels (Taqman) in the subset of 13 primary tumors for which clinically obtained Hercept test data and adequate RNA were available (Table 4). Consistent with the previously reported frequency of ERBB2 overexpression in primary breast tumors of approximately 30% (reviewed in [2]), four of these 13 primary tumors had a positive Hercep test (2+ or 3+) reported by a clinical lab. The two tumors with 3+ staining intensity also had IR >2 for the BAC clone closest to ERBB2 on the CGH array (RP11-552K3) and had a Q-PCR relative ratio >1.5, validating the aCGH data. These tumors also showed overexpression of *ERBB2 *mRNA compared to regions without copy number increase, with a TaqMan ratio >5.

### Pathway mapping of aCGH data

**Figure 3:**
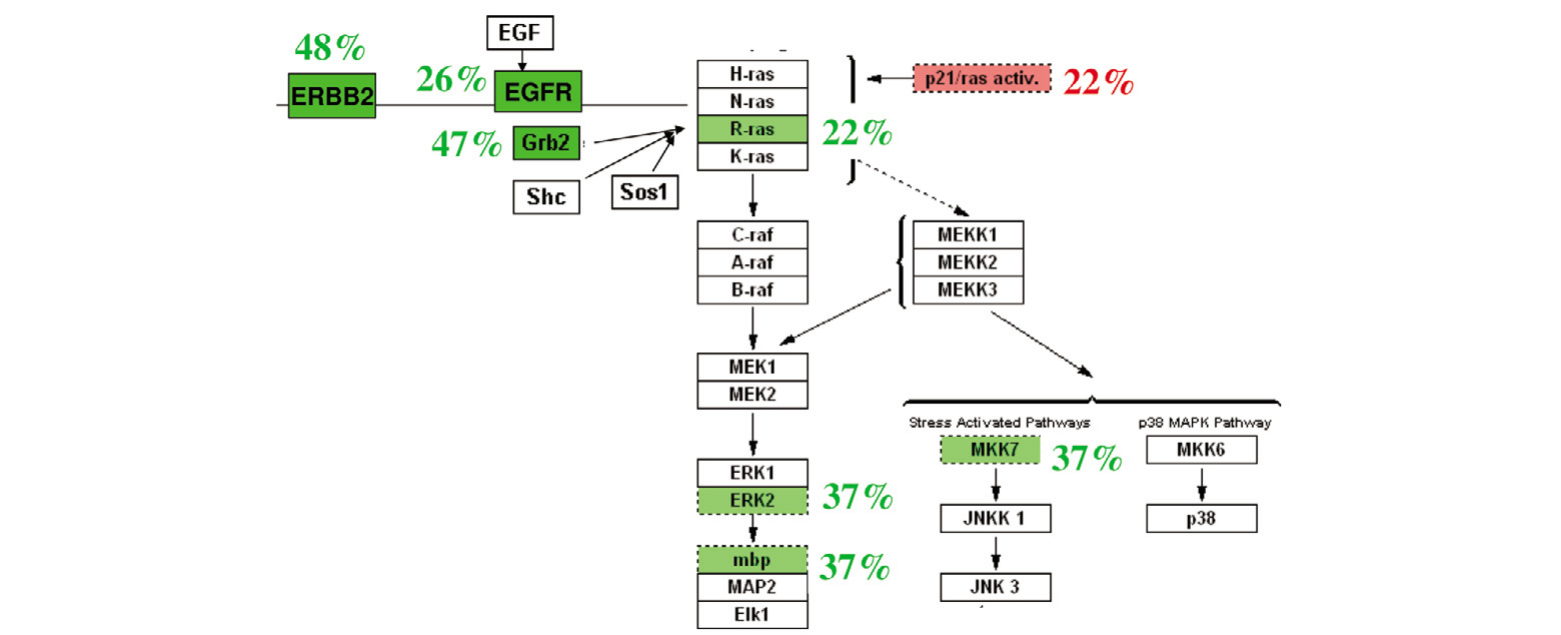
Genmapp view of the epidermal growth factor (EGF) signaling pathway. Array-based comparative genomic hybridization copy number frequency data are represented with gains in green and losses in red.

As noted above, the EGF/ERBB2 signaling pathway is clinically relevant in breast cancer. We thus used GenMapp [21] to visualize a composite analysis of DNA copy number of this pathway (Fig. 3). Of note, one or more genes in the canonical EGF signaling pathway were altered in 39/47 primary tumors (83%). Three genes in this pathway had IRs >2 in our set of 47 primary tumors; *EGFR *in two tumors, *ERBB2 *in three tumors, and *GRB2 *in one tumor. A hemizygous deletion of *RASA1*, which encodes the Ras-GAP that deactivates H-Ras, was detected in four tumors.

### Cooperating genetic loci

**Figure 4:**
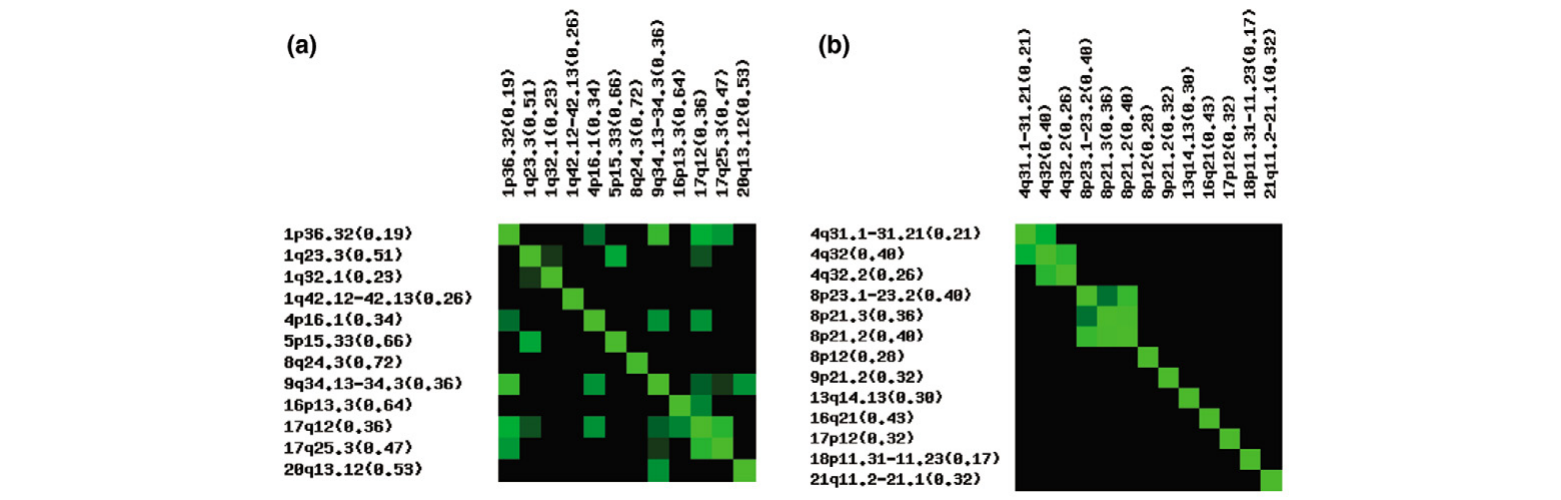
Correlations between gained and lost loci in breast cancer. Correlation matrices depicting the interaction between the most frequently gained **(a) **and lost **(b) **loci in our dataset. Black indicates a p-value > 0.05, whereas the green color becomes more intense as the p-value decreases. Cytoband location is indicated, along with the frequency of aberration in parentheses.

As a means of identifying genetic alterations that may function coordinately in tumor initiation and progression, we looked for correlation between genetic loci (i.e. for genes that were gained or lost coordinately with other genes more commonly than would be expected by chance). Separate correlation matrices (Fig. 4) were created using the most commonly altered loci (Table 1). Full heatmaps for genes and loci are available online [22]. Each matrix was evaluated for positive correlation (concordant gains or losses) and negative correlation (discordant gains or losses). To account for the large number of comparisons, we determined the significance of the correlation between these loci using the Fisher's exact test to generate a p-value, again with gains and losses considered separately.

Although several positive correlations were identified, negative correlations with p < 0.05 were not found. With the exception of loci on the same chromosome, only one pair of common losses was correlated, those on chromosome 4:141.6–145.2 Mb and chr13: 44.6–45.1 Mb (p = 0.004). In contrast, common regions of gain were highly correlated to one another. Chromosome 1 and 9 showed the strongest correlation (p < 0.0001), but these loci are also significantly correlated to many of the other commonly gained loci in the matrix. One exception is a frequent gain on chromosome16, which is not correlated to either of these loci, but is highly correlated to chromosome 17q12 (p = 0.0001).

## Discussion

aCGH is a powerful technique that allows determination of DNA copy number across the genome of a tumor in a single experiment, with resolution limited only by the number of elements on the array. As a result, aCGH detects changes at higher frequency and with smaller CROs than previous approaches (i.e., chromosomal CGH). Finally, aCGH is semiquantitative, providing an opportunity to narrow regions of copy number gain to those genes most likely to be biologically significant by identifying infrequent high-level amplifications in regions of frequent, lower copy number gain, analogous to using homozygous deletions to narrow larger regions of hemizygous loss.

Perhaps the most surprising finding of this study is the very similar pattern of gains and losses in primary tumors and cell lines. Although there are significantly more frequent genomic alterations in the cell lines, the pattern of gain and loss is strikingly similar to that seen in the primary tumors. It has long been thought that cell lines contained substantial amounts of genomic noise – random, biologically insignificant copy number alterations considered a reflection of the inherent genomic instability of human cancers. The current data argue to the contrary, suggesting that the amplification and deletions seen in cell lines offer the same growth advantages in cell culture that they do *in vivo*, and are similarly selected for over multiple passages. Furthermore, the relative concordance of genomic aberrations in cell lines and primary tumors increases the confidence in the former as relevant *in vitro *models and should in the near future allow a direct assessment of how closely a given cell line reflects the parent genotype from which it was derived. Finally, these data also suggest that cancer genomes are relatively stabile over time, unlike expression profiles, which can vary dramatically in short periods of time in response to various growth conditions.

Another surprising finding is the lack of correlation between prognostic clinical parameters such as stage, grade, and receptor status and the overall frequency of genome copy number alterations. A relationship between estrogen receptor status and both overall genomic aberrations as well as specific regions of common gains and losses has been recently reported [14]. Although our data do not confirm their findings, we cannot exclude the possibility that the relatively small sample size of this study provided limited power to see these correlations; however, similar results were recently reported with an aCGH analysis of bladder cancers [23]. If validated in larger series, these data suggest that it is the specific genetic changes, not the total number of copy number alterations, that are determinants of outcome. This hypothesis is consistent with global expression profiling data in breast cancers, where altered expression of a small subset of genes in the human genome was shown to be predictive of outcome years after diagnosis [24]. A larger dataset with longer follow-up than the current sample set is needed to determine whether a subset of copy number alterations will be similarly predictive of long term outcome. As expression profiles are a reflection of the fixed genetic changes in cancer genomes, however, it is likely that aCGH profiles of specific gene sets will be similarly predictive. This would be of great clinical utility, as aCGH may be performed on archival material, which is much more readily available than the frozen tumor currently required for expression profiling. Coupled with the likelihood that fixed genetic abnormalities in cancer genomes may be more predictive of response to specific therapy, aCGH holds significant promise for clinical benefit.

Analysis of some of the most commonly altered regions in this sample set illustrate the complex pattern of copy number change that can be clarified with aCGH. For example, the 8q24 amplicon has previously been attributed *MYC*; however, aCGH of this sample set detects two distinct regions of amplification, with the most frequent region of gain being more telomeric to that which includes *MYC*. Expression profiling of breast and other epithelial cancers similarly suggested that *MYC *is less frequently overexpressed than genes located closer to the 8q telomere [25,26]. *PTK2 *(*FAK*) falls within this more telomeric region. *PTK2 *encodes a cytoplasmic tyrosine kinase, central to several proliferative pathways, including integrin, G-protein coupled, and receptor tyrosine kinase signaling, and thus has a plausible role in cancer biology [27]. Consistent with this hypothesis, breast cancer cell lines grown in monolayer culture frequently express constitutively activated *PTK2*, whereas normal mammary epithelial cells grown under similar conditions do not [28]. In fact, copy number gains of *PTK2 *are frequent in cell lines derived from invasive epithelial tumors, and *PTK2 *amplification correlates with increased protein expression in squamous carcinoma cell lines [29].

In addition to increasing the mapping resolution of known regions of copy number change, aCGH is an unbiased approach to detecting novel regions of genomic alteration, which potentially harbor novel cancer-related genes. For example, these data reveal several novel amplicons on chromosome 1, as well as a narrow region of high-level gain at 1q32.1, which is commonly gained in breast cancer. This region includes *CNTN2*, *RBBP5*, *ELK4*, *Prostein*, *NUCKS *and two hypothetical genes. Expression profiling of a subset of these tumors demonstrate that *NUCKS *and two ESTs (Expressed Sequence Tags) are overexpressed in the tumors with amplifications relative to tumors that are diploid at this locus. Evidence that *NUCKS *(nuclear ubiquitous casein kinase and cyclin-dependent kinase substrate) is expressed in breast tissue and is believed to play a role in regulating transcriptional regulation makes it an excellent candidate gene in this region [30]. The increased resolution of aCGH also revealed several small regions of modest copy number gain or hemizygous deletion that have not been associated with breast tumors in previous studies. Gains at the telomeres of 4p (4p16.1) and 5p (5p15.33) are examples. While 4p16.1 does not contain any known cancer-related genes, the catalytic unit of telomerase (*hTERT) *is contained within the 5p15.33 amplicon.

The current data also suggest that homozygous deletions are relatively uncommon in primary tumors. The only homozygous deletion we detected in the primary tumors is on chromosome 9p21. This region contains *MOB3B*, which shares similarity with the yeast gene scMob1. scMob1 binds Mps1p, a protein kinase essential for spindle pole body duplication and mitotic checkpoint regulation, which in turn plays a role in maintaining genome stability, again providing biological plausibility for loss of *MOB3B *in cancer. Homozygous deletions are more common in cell lines, suggesting positive selection for loss of the genes in these regions and possibly an increased tolerance to the loss of adjacent genes in immortalized cell lines. An alternative explanation may be that contamination by normal (diploid) cells in primary tumors might decrease the sensitivity of detection of homozygous losses compared to homogenous cell lines. As an example, the recurrent homozygous deletion on 18q21 (46.8–52.8 Mb), which includes *SMAD4 *(*MADH4*), also has been observed only in pancreatic cell lines [31]. Another region of frequent hemizygous loss that contains a recurrent homozygous deletion in cell lines is 8p23. Although this region contains 14 genes, 12 of them belong to the defensin family. Interestingly, defensins play a role in epithelial wound repair, which involves migration, proliferation and *EGFR *activation [32].

A comparison of the relative frequency of gains and losses at specific loci may provide insight not only into the likelihood that change at a specific locus is of biological significance, but into the biological function of the associated genes as well. For example several tumor suppressor genes known to play a role in breast cancer, such as *RB1*, *PTEN *and *BRCA2*, were frequently lost, but rarely gained, in our data set. Conversely, most kinases were gained on average five times more frequently than they were deleted. These data suggest that regions that are equally likely to be gained and lost are unlikely to contain genes that confer a selective advantage when altered. As an example, analysis of copy number changes in kinases, expected to be amplified in cancers, revealed an interesting relationship between two members of the focal adhesion kinase family. *PTK2 *(*FAK*) is the most frequently gained gene overall and *PTK2B *(*PYK*) is the most frequently lost kinase. Interestingly, these gene products differentially regulate progression of the cell cycle, with induction of *PTK2B *inhibiting G1-S transition, while induction of *PTK2 *expression increases the rate of this transition [33].

Finally, we have used two different methods, pathway mapping and correlation analysis, to interrogate the aCGH data for evidence of interaction between genomic loci. In this instance, pathway mapping suggests that only a subset of the genes in a pathway may confer a selective advantage when altered in a specific tissue type. Thus *ERBB2*, *EGFR *and *GRB2 *were frequently amplified, but *RAS*, *RAF *and *MEK *were amplified in less than 10% of the tumors. Many tumors had more than one copy number alteration in this pathway, but none had a high level amplification in more than one node. Only *RASA1*, which encodes a Ras-GAP that deactivates H-Ras, was hemizygously deleted in this pathway. One of the tumors with a *RASA1 *deletion did not have a gain in any of the activating genes in this pathway, suggesting a mechanism of H-Ras activation in this tumor.

The correlation analysis demonstrated both specific loci that may cooperate in initiating or maintaining the malignant phenotype and some generalized differences between gains and losses. These observations are consistent with the hypothesis that gains and losses in cancer genomes are generated by different mechanisms and subject to different selection. Thus, when compared to deletions, gains are more common, often larger, and much more frequently correlated with other gains. One model of genomic instability that is consistent with these findings is large scale duplication of the entire genome with subsequent loss of whole chromosomes or smaller intrachromosomal regions.

The relatively small numbers of patients in each clinical subgroup did not allow us to identify association between specific aberrations and clinical characteristics at this time; this may require both more samples as well as novel analytical methods to analyze patterns of similar aberrations. In addition to clinical and histological characteristics, expression profiling is emerging as a viable means of molecularly subtyping breast cancer, and a recent report has correlated distinct regions of loss of heterozygosity with specific expression profiles [34]. Further work will be required to realize the potential of combining data from expression analysis and CGH to pinpointing genes affected by amplifications and deletions, which should lead to both a better understanding of the significance of specific genetic aberrations as well as novel targets for therapeutic interventions.

## Conclusion

A set of primary breast cancers and breast cancer cell lines subjected to aCGH were found to be strikingly similar to one another and together they generated a rich dataset for inquiry into the cancer genome. Findings from the analysis of these data include fine mapping of previously described regions of gain and loss, identification of novel regions of gain and loss, and unbiased enumeration of the frequency of copy number alterations in specific genes. This analysis also was applied to gene subsets, including kinases and tumor suppressor genes, and specific pathways, which not only provided a rank order list of the most common alterations but had apparent functional implications. Finally, correlation analysis identified specific potential cooperating loci and highlighted possible differences in genomic mechanisms that generate gains and losses. These findings require additional investigation but have the potential to be of substantial biological and therapeutic significance.

## Abbreviations

aCGH = array-based comparative genomic hybridization; BAC = bacterial artificial chromosome; CGH = comparative genomic hybridization; CRO = common region of overlap; EGF = epidermal growth factor; IDC = infiltrating ductal carcinoma; ILC = infiltrating lobular carcinoma; IR = intensity ratio; Mb = megabase; Q-PCR = quantitative PCR.

## Competing interests

The authors declare that they have no competing interests.

## Authors' contributions

TN carried out aCGH, gene expression and Q-PCR studies, analyzed these data and drafted the manuscript. JG performed statistical analysis, generated correlation plots, and helped with data analysis. VC was the surgeon who provided the breast tumor specimens as well as access to the clinical data associated with these surgeries. QY and YW performed the pathology analysis for each tumor and prepared the samples for nucleic acid extraction. TZ helped draft the manuscript. BW conceived of the study, participated in its design and coordination and helped draft the manuscript. All authors read and approved the final manuscript.

## Supplementary Material

Additional File 1 Table listing the clinical data and tumor characteristics for primary tumors. Click here for file

Additional File 2 Table lisitng all regions of copy number gain present in ≥ 30% of primary breast tumors. Click here for file

Additional File 3 Table showing the lack of correlation between clinical characteristic and amount of genomic copy number change. Click here for file

Additional File 4 Table showing the top 10 gains and losses in primary tumors and cell lines organized by gene class. Click here for file

## Figures and Tables

**Table 1:** Frequent DNA copy number alterations in breast cancer: previous reports compared with the current study

Genomic change	Previous reports			Current study					
	Reference	Region	% Tumors	Cytoband^a^	Start (Mb)^a,b^	Stop (Mb)^a,b^	Size (Mb)^a,b^	% Tumors	Cancer genes

Gains									
1q+				1p36.32	2.0	6.0	4.0	49	
1q+				1q23.3	158.8	159.6	0.8	57	
1q+	[5-10]	1q32	48–67	1q32.1	202.1	202.9	0.8	66	
1q+				1q42.12-42.13	221.8	225.9	4.1	53	
4p+				4p16.1	7.1	8.8	1.7	51	
5p+	[6]	5p12-14	24	5p15.33	0	1.9	1.9	49	*TERT*
8q+	[5-10]	8q22-qter	39–49	8q24.3	139.3	144.8	5.6	79	*PTK2*
9q+				9q34.13-34.3	130.5	135.2	4.6	53	*TSC1 RALGDS*
16p+	[6]	16p	38–40	16p13.3	3.2	3.3	0.1	57	*MMP25*
17q+	[6-10]	17q12	18–60	17q12	33.6	38.9	5.3	45	*TAF15 MLLT6 ERBB2*
17q+	[5-10]	17q22-25	18–60	17q25.3	77.7	81.1	3.5	45	*GRB2 TIMP2*
20q+	[5-10]	20q13	18–44	20q13.12	43.8	45.7	1.9	49	*MMP9*
									
Losses									
4q-				4q31.1-31.21	141.6	145.2	3.6	40	
4q-				4q32	156.1	160.1	4.0	36	
4q-				4q32.2	162.9	164.2	1.3	32	
8p-	[7]	8p22-23	20	8p23.1-23.2	4.8	7.6	2.8	40	
8p-	[6,8,10]	8p	18–29	8p21.3	19.9	20.0	0.1	32	
				8p21.2	23.5	27.5	4.0	36	*PTK2B*
				8p12	34.8	35.6	0.8	32	
9p-	[6,7,9]	9p22-24	20–23	9p21.2	27.6	27.9	0.3	30	
13q-	[6-8,10]	13q21-31; 13q22-31	18–57	13q14.13	44.6	45.1	0.5	30	*LCP1*
16q-	[6-8]	16q21-qter; 16q23-24	20–38	16q21	61.5	63.5	2.0	34	*CDH8*
17p-				17p12	11.5	11.6	0.1	32	
18p-				18p11.31-11.23	6.1	7.6	1.5	36	
21q-				21q11.2-21.1	15.3	15.9	0.6	32	

Regions of gain and loss identified in previous studies are shown in comparison to those regions as detected in the present study. ^a^Map positions and cytogenetic locations are based on data available from UCSC genome browser (July 2003 freeze) [35]. ^b^Region boundaries were determined by the next bacterial artificial chromosome with different copy number.

**Table 2:** Regions of high-level copy number gain

Chromosome	Region (Mb)^a,b^	Cell lines IR > 2	Tumors IR > 2	Tumors IR 1.2–2.0	Cancer genes
1	158.8–159.6	0	2 (4%)	29 (61.7%)	
1	202.1	4 (22%)	4 (9%)	31 (66.0%)	*KISS1*
4	6.9	1 (6%)	2 (4%)	22 (46.8%)	
6	11.2–15.6	0	2 (4%)	(%)	
6	139.6–143.6	0	2 (4%)	(%)	
7	54.4–57.1	2 (11%)	2 (4%)	12 (25.5%)	*EGFR*
8	0.1–0.3	1 (6%)	2 (4%)	31 (66.0%)	
8	36.9–42.0	1 (6%)	4 (9%)	13 (27.7%)	*WHSCIL1*, *FGFR*
8	74.0–75.1	1 (6%)	2 (4%)	11 (23.4%)	
8	123.8–127.5	3 (17%)	2 (4%)	20 (42.6%)	*MYC*
8	139.3–144.8	10 (56%)	5 (11%)	37 (78.7%)	*PTK2*
11	58.8–61.3	0	3 (6%)		
11	66.1–71.5	1 (6%)	2 (4%)		*DDB1*
11	73.4–77.5	1 (6%)	2 (4%)		*WNT11*, *PAK1*
13	111.4–112.9	4 (22%)	4 (9%)	21 (44.7%)	*GAS6*
17	35.1–38.3	2 (11%)	5 (11%)	17 (36.2%)	*TAF15*, *MLLT6*, *ERBB2*
17	58.1–60.4	2 (11%)	2 (4%)	10 (21.3%)	*PPM1D*
20	43.8–45.9	4 (22%)	3 (6%)	5 (10.6%)	*MMP9*
20	49.2–50.0	2 (11%)	2 (4%)		
21	45.1–45.8	3 (17%)	5 (11%)	18 (38.3%)	*ITGB2*

Regions of copy number gain containing more than one amplification with intensity ratio (IR) >2.0, as well as frequency of single copy gain (IR 1.2–2.0) in these regions. ^a^Map positions and cytogenetic locations are based on data available from UCSC genome browser (July 2003 freeze) [35]. ^b^Region boundaries were determined by the next bacterial artificial chromosome with different copy number.

**Table 3:** Regions containing a putative homozygous deletion in more than one cell line

Chromosome	Region (Mb)^a,b^	CRO (Mb)^a,b^	Cell lines with homozygous loss	Tumors with hemizygous loss	Genes
8	4.8–7.6	2.8	3 (16.7%)	19 (40%)	*ANGPT2*, *LPAAT-e*, *DEFA1*, *DEFA3*, *DEFA4*, *DEFA5*, *DEFA6*, *DEFB1*, *DEFB10*, *DEFT1*, *SPAG11*, *DEFB103*, *DEFB104*, *DEFB105*, *DEFB106*, *DEFB4*, *CLDN23*
8	10.3–11.7	1.4	2 (%)	6 (13%)	*UNQ9391*, *RP1L1*, *SOX7*, *PINX1*, *MTMR9*, *AMAC*, *TDH*, *BLK*, *GATA4*, *NEIL2*
8	19.9–20.0	0.1	2 (11.1%)	15 (32%)	*PCM1*, *NAT1*, *NAT2*, *EFA6R*, *ChGn*, *LPL*, *GFRA2*, *DOK2*, *XPO7*, *NPM2*, *FGF17*, *EPB49*, *RAI16*
8	23.5–27.5	4.0	2 (11.1%)	17 (36%)	*TNFRSF10A*, *EFL*, *DOCK5*, *GNRH1*, *KCTD9*, *EBF2*, *PPP2R2A*, *BNIP3L*, *PTK2B*
9	0.6–4.2	3.6	2 (11.1%)	9 (19%)	*ANKRD15*, *DMRT1*, *DMRT2*, *DMRT3*, *SMARCA2*, *VLDLR*, *KCNV2*, *RFX3*, *ZNF515*
9	27.6–27.9	0.3	2 (11.1%)	14 (30%)	*ELAV*, *PLAA*, *CCDC2*, *LRRC19*, *TEK*, *MOB3B*, *IFNK*
13	72.1–74.0	1.9	2 (11.1%)	6 (13%)	*KLF12*, *TBC1D4*, *UCHL3*
18	32.2–37.4	5.2	2 (11.1%)	9 (19%)	*BRUNOL4*
18	38.1–41.8	3.7	3 (16.7%)	13 (28%)	*RIT2*, *SYT4*, *SETBP1*
18	46.8–52.8	6.0	2 (11.1%)	6 (13%)	*MADH4*, *DCC*, *MBD2*, *POLI*, *STARD6*, *RAB27B*, *SE57-1*, *TCF4*, *TXNL*, *WDR7*

^a^Map positions and cytogenetic locations containing putative homozygous deletions (intensity ratio <0.5 within a region of hemizygous loss) are based on data available from UCSC genome browser (July 2003 freeze) [35]. ^b^Region boundaries were determined by the next bacterial artificial chromosome with different copy number. CRO, common region of overlap.

**Table 4:** Array-based comparative genomic hybridization validation by DNA quantitative PCR of *ERBB2*

Tumor ID	DNA				RNA	Protein
	aCGH RP11-552K3		Light cycler		TaqMan	Hercept

	Cy3	Cy5	Relative ratio	Relative alleles	Ratio	Staining

40	1.05	1.13	0.67	1	0.51	-
71	1.28	0.58	0.94	2	2.01	-
88	1.48	0.45	1.08	2	2.08	-
268	1.51	0.76	0.94	2	1.78	-
313	0.99	1	0.82	2	0.95	-
348	1.06	0.8	0.88	2	0.99	-
352	1.64	0.76	0.88	2	n.d.*	-
476	1.03	0.88	0.94	2	1.72	-
493	1.25	0.97	0.88	2	n.d.	-
277	1.14	0.99	0.94	2	0.67	2+
445	1.42	0.87	0.88	2	0.43	2+
316	2.41	0.43	1.51	3	5.61	3+
369	2.45	0.29	3.88	8	16.1	3+

*ERBB2 *validation of array-based comparative genomic hybridization (aCGH) DNA copy number using LightCycler Her2/Neu DNA quantification kit (Roche), TaqMan Assay on Demand Hs. 00170433_m1 (Applied Biosystems), and protein quantification (Herceptest^®^). * n.d.: Not done
