# Supplementary material for: High resolution genomic analysis of sporadic breast cancer using array-based comparative genomic hybridization
Source: Breast Cancer Res. 2005 Nov 24;7(6):R1186–98. doi: 10.1186/bcr1356 (PMC1410746; doi:10.1186/bcr1356)
Supplement: Additional File 1 — Table listing the clinical data and tumor characteristics for primary tumors. [file bcr1356-S1.doc]

Supplementary Data Table 1 – **Clinical data and tumor characteristics for primary tumors.**

| Sample | Age | Dx | ER status | PR status | Her2 status | Ploidy | Stage | Recurrence |
| --- | --- | --- | --- | --- | --- | --- | --- | --- |
| TB0001 | 51 | IDC | Negative | Negative | Positive | Aneuploid | IIIB |  |
| TB0003 | 53 | IDC | Negative | Negative | Positive | Aneuploid | IIB | local |
| TB0004 | 41 | IDC | Positive | Positive | Positive | Aneulpoid | IIIC |  |
| TB0005 | 44 | IDC | Negative | Negative | Positive | Aneuploid | IIIC |  |
| TB0007 | 36 | IDC | Positive | Positive | Positive | Diploid | I |  |
| TB0010 | 50 | IDC | Positive | Positive |  | Aneuploid | IV | distant |
| TB0016 | 74 | IDC | Positive | Positive |  | Diploid |  |  |
| TB0017 | 34 | MIXED | Negative | Negative | Negative | Diploid | IIIB |  |
| TB0020 | 40 | IDC |  |  |  |  |  |  |
| TB0022 | 69 | IDC | Negative | Negative | Negative | Aneuploid | IIA | local |
| TB0026 | 70 | IDC | Positive | Positive | Positive | Aneuploid | IIA | local |
| TB0029 | 37 | IDC | Negative | Negative | Negative | Aneuploid | I |  |
| TB0031 | 54 | IDC | Positive |  | Negative |  | IV | distant |
| TB0032 | 51 | IDC | Negative | Negative |  | Aneuploid | IIIA |  |
| TB0033 | 62 | IDC | Positive | Positive | Negative | Aneuploid | IIIC |  |
| TB0036 | 79 | MIXED |  |  |  |  |  | local |
| TB0038 | 52 | ILC | Positive | Positive | Negative | Aneulpoid | IIA |  |
| TB0040 | 49 | IDC | Positive | Negative | Negative | Aneuploid | IIB |  |
| TB0041 | 50 | IDC | Negative | Negative | Negative | Aneuploid | IV | distant |
| TB0046 | 52 | IDC | Negative | Negative | Positive | Aneuploid | IIA |  |
| TB0054 | 49 | MIXED | Positive | Positive |  | Diploid | IIA |  |
| TB0055 | 80 | IDC | Negative | Negative | Negative | Aneuploid | IIA |  |
| TB0063 | 51 | IDC | Positive | Positive | Negative | Aneuploid | I | local |
| TB0064 | 67 | IDC | Positive | Positive |  | Diploid | IIIB |  |
| TB0065 | 55 | DCIS | Borderline | Negative | Positive | Aneuploid | IV | distant |
| TB0071 | 94 | MIXED | Positive | Positive | Negative | Diploid | IIIB |  |
| TB0072 | 50 | IDC | Negative | Negative | Negative | Aneuploid | I |  |
| TB0088 | 49 | IDC | Positive | Positive | Negative | Aneuploid | IIB |  |
| TB0268 | 38 | IDC | Negative | Negative | Negative | Aneuploid | I |  |
| TB0275 | 97 | IDC | Positive | Negative | Negative | Aneulpoid | IIIB |  |
| TB0277 | 45 | IDC | Negative | Negative | Positive | Diploid | IIA |  |
| TB0313 | 85 | IDC | Negative | Negative | Negative | Aneuploid | IIA |  |
| TB0314 | 60 | IDC | Positive | Positive | Positive | Aneuploid | IIIA | local |
| TB0315 | 41 | IDC | Negative | Negative | Negative | Anueploid | IIB | local |
| TB0316 | 43 | IDC | Negative | Negative | Positive |  | IIIA |  |
| TB0318 | 68 | DCIS | Positive | Negative | Negative |  | I |  |
| TB0344 | 38 | IDC | Positive | Positive | Negative | Aneuploid | IIIC |  |
| TB0346 | 47 | IDC | Positive | Positive | Positive | Aneuploid | IIB |  |
| TB0348 | 80 | IDC | Positive | Negative | Negative | Diploid | I |  |
| TB0352 | 87 | IDC | Positive | Positive | Negative | Diploid | I |  |
| TB0369 | 36 | IDC | Positive | Negative | Positive | Aneuploid | IIB |  |
| TB0432 | 62 | IDC | Positive | Positive | Positive | Aneuploid | I | local |
| TB0433 | 79 | ILC | Positive | Positive | Positive | Diploid | IV | distant |
| TB0445 | 44 | IDC | Positive | Positive | Positive | Diploid | I |  |
| TB0455 | 44 | IDC | Negative | Negative | Negative | Diploid | IIB |  |
| TB0456 | 30 | IDC | Positive | Positive | Positive |  | IIIB |  |
| TB0476 | 74 | IDC | Positive | Positive | Negative | Diploid | IIB |  |
| TB0482 | 57 | IDC |  |  |  |  | IIB |  |
| TB0493 | 52 | IDC | Negative | Negative | Negative | Diploid | IV | distant |
