# Supplementary material for: High resolution genomic analysis of sporadic breast cancer using array-based comparative genomic hybridization
Source: Breast Cancer Res. 2005 Nov 24;7(6):R1186–98. doi: 10.1186/bcr1356 (PMC1410746; doi:10.1186/bcr1356)
Supplement: Additional File 2 — Table lisitng all regions of copy number gain present in ≥ 30% of primary breast tumors. [file bcr1356-S2.doc]

Supplementary Data Table 2 – **All regions of copy number gain present in ≥30% of primary breast tumors.**
