# Supplementary material for: High resolution genomic analysis of sporadic breast cancer using array-based comparative genomic hybridization
Source: Breast Cancer Res. 2005 Nov 24;7(6):R1186–98. doi: 10.1186/bcr1356 (PMC1410746; doi:10.1186/bcr1356)
Supplement: Additional File 3 — Table showing the lack of correlation between clinical characteristic and amount of genomic copy number change. [file bcr1356-S3.doc]

Supplementary Data Table 3 – **Lack of correlation between clinical characteristic and amount of genomic copy number change.** Total genomic copy number change expressed as the percent of the genome that is aberrant (sum of gains and losses). ^a^ The Wilcoxon rank sum test was used to generate the p-value for the comparisons between groups. *Histological grade, **Abstracted from patient questionnaire at diagnosis

|  | N | Total Genomic Change | | | Gain | | | Loss | | |
| --- | --- | --- | --- | --- | --- | --- | --- | --- | --- | --- |
|  |  | mean | stddev | p-value^a^ | mean | stddev | p-value^a^ | mean | stddev | p-value^a^ |
| Cell Lines | 18 | 44.8 | 15.3 | 0.00 | 24.0 | 8.6 | 0.00 | 20.8 | 8.0 | 0.00 |
| Primary Tumors | 42 | 24.6 | 14.5 |  | 14.5 | 9 |  | 10.1 | 6.9 |  |
|  |  |  |  |  |  |  |  |  |  |  |
| Stage I | 10 | 26.8 | 13.6 | 0.79 | 14.9 | 6.2 | 0.76 | 11.8 | 8.6 | 0.85 |
| Stage II | 16 | 22.6 | 12.6 |  | 13.2 | 7.7 |  | 9.5 | 5.8 |  |
| Stage III | 9 | 23.6 | 11.1 |  | 14.4 | 6.7 |  | 9.2 | 6.8 |  |
| Stage IV | 5 | 21.4 | 16.2 |  | 12.1 | 7.6 |  | 9.3 | 9.0 |  |
|  |  |  |  |  |  |  |  |  |  |  |
| Stage I+II+III | 35 | 24.1 | 12.3 | 0.47 | 14.0 | 6.9 | 0.47 | 10 | 6.8 | 0.61 |
| Stage IV | 5 | 21.4 | 16.2 |  | 12.1 | 7.6 |  | 9.4 | 9.1 |  |
|  |  |  |  |  |  |  |  |  |  |  |
| Grade 1^*^ | 2 | 12.6 | 9.1 | 0.12 | 5.8 | 1.6 | 0.10 | 6.7 | 7.5 | 0.23 |
| Grade 2 | 14 | 27.1 | 11.6 |  | 15.0 | 6.1 |  | 12.1 | 7.3 |  |
| Grade 3 | 24 | 21.2 | 12.7 |  | 12.7 | 6.9 |  | 8.6 | 6.8 |  |
|  |  |  |  |  |  |  |  |  |  |  |
| ERBB2 Negative | 25 | 25.1 | 13 | 0.48 | 14.5 | 7 | 0.28 | 10.6 | 7.4 | 0.69 |
| ERBB2 Positive | 11 | 21.1 | 12 |  | 11.7 | 5.7 |  | 9.5 | 7.1 |  |
|  |  |  |  |  |  |  |  |  |  |  |
| ER Negative | 13 | 23.3 | 13.7 | 0.23 | 13.3 | 6.4 | 0.32 | 10.1 | 8.4 | 0.28 |
| ER Positive | 25 | 23.6 | 11.1 |  | 14.0 | 6.9 |  | 9.6 | 5.7 |  |
|  |  |  |  |  |  |  |  |  |  |  |
| Premenopausal^**^ | 19 | 23.3 | 12.1 | 0.89 | 14.3 | 5.9 | 0.32 | 9 | 7.7 | 0.29 |
| Postmenopausal | 23 | 23.4 | 13 |  | 12.8 | 7.6 |  | 10.6 | 6.3 |  |
|  |  |  |  |  |  |  |  |  |  |  |
| Diploid | 13 | 22 | 11 | 0.70 | 13.7 | 7.7 | 0.94 | 8.3 | 4.3 | 0.61 |
| Aneuploid | 25 | 24 | 13.1 |  | 13.6 | 6.6 |  | 10.4 | 7.7 |  |
