# Supplementary material for: High resolution genomic analysis of sporadic breast cancer using array-based comparative genomic hybridization
Source: Breast Cancer Res. 2005 Nov 24;7(6):R1186–98. doi: 10.1186/bcr1356 (PMC1410746; doi:10.1186/bcr1356)
Supplement: Additional File 4 — Table showing the top 10 gains and losses in primary tumors and cell lines organized by gene class. [file bcr1356-S4.doc]

Supplementary Data Table 4 – **Top 10 gains (a) and losses (b) in primary tumors and cell lines organized by gene class.**

| (a) Top 10 GAINS | |  |  |  |  | (b) Top 10 LOSSES | |  |  |  |
| --- | --- | --- | --- | --- | --- | --- | --- | --- | --- | --- |
| Tumor Suppressor Genes^1^ | Gain Primary Tumors | Loss Primary Tumors | Gain Cell Lines | Loss Cell Lines |  | Tumor Suppressor Genes^1^ | Loss Primary Tumors | Gain Primary Tumors | Loss Cell Lines | Gain Cell Lines |
| *BRCA1* | 34.0% | 2.1% | 44.4% | 0.0% |  | *RB1* | 25.5% | 0.0% | 50.0% | 5.6% |
| *EXT1* | 31.9% | 2.1% | 77.8% | 0.0% |  | *CYLD* | 25.5% | 2.1% | 61.1% | 5.6% |
| *TSC1* | 31.9% | 2.1% | 44.4% | 0.0% |  | *MAP2K4* | 23.4% | 2.1% | 27.8% | 5.6% |
| *EP300* | 29.8% | 4.3% | 38.9% | 5.6% |  | *PTCH* | 19.1% | 4.3% | 33.3% | 11.1% |
| *TSC2* | 29.8% | 0.0% | 22.2% | 0.0% |  | *CDKN2A* | 17.0% | 0.0% | 38.9% | 0.0% |
| *NF2* | 27.7% | 2.1% | 38.9% | 0.0% |  | *APC* | 12.8% | 2.1% | 22.2% | 16.7% |
| *SMARCB1* | 27.7% | 2.1% | 55.6% | 0.0% |  | *MLH1* | 12.8% | 0.0% | 44.4% | 5.6% |
| *CDH1* | 21.3% | 4.3% | 61.1% | 11.1% |  | *PTEN* | 10.6% | 0.0% | 38.9% | 5.6% |
| *MEN1* | 19.1% | 4.3% | 38.9% | 0.0% |  | *BRCA2* | 8.5% | 0.0% | 27.8% | 0.0% |
| *STK11* | 12.8% | 6.4% | 16.7% | 16.7% |  | *MSH2* | 8.5% | 4.3% | 16.7% | 16.7% |
| Kinases^2^ |  |  |  |  |  | Kinases^2^ |  |  |  |  |
| *PTK2* | 70.2% | 0.0% | 72.2% | 11.1% |  | *PTK2B* | 34.0% | 6.4% | 66.7% | 0.0% |
| *SNARK* | 59.6% | 2.1% | 77.8% | 0.0% |  | *PHKB* | 27.7% | 2.1% | 38.9% | 11.1% |
| *ITPKB* | 53.2% | 0.0% | 72.2% | 0.0% |  | *DCAMKL1* | 27.7% | 0.0% | 44.4% | 5.6% |
| *STK4* | 48.9% | 0.0% | 72.2% | 0.0% |  | *TEK* | 25.5% | 0.0% | 55.6% | 0.0% |
| *AKT2* | 46.8% | 2.1% | 61.1% | 0.0% |  | *MAP2K4* | 23.4% | 2.1% | 27.8% | 5.6% |
| *MAP3K10* | 46.8% | 2.1% | 61.1% | 0.0% |  | *CSNK2A2* | 29.8% | 25.5% | 55.6% | 27.8% |
| *PCTK3* | 46.8% | 0.0% | 66.7% | 0.0% |  | *BCKDK* | 27.7% | 8.5% | 22.2% | 33.3% |
| *RHOK* | 44.7% | 6.4% | 66.7% | 5.6% |  | *MAPK3* | 27.7% | 8.5% | 22.2% | 33.3% |
| *MYLK2* | 44.7% | 2.1% | 66.7% | 0.0% |  | *PHKG2* | 27.7% | 8.5% | 22.2% | 33.3% |
| *CSNK1D* | 42.6% | 4.3% | 61.1% | 0.0% |  | *TAO1* | 27.7% | 8.5% | 22.2% | 33.3% |
| Cancer Genes^3^ | |  |  |  |  | Cancer Genes^3^ | |  |  |  |
| *PTK2* | 70.2% | 0.0% | 72.2% | 11.1% |  | *PTK2B* | 34.0% | 6.4% | 66.7% | 0.0% |
| *SNARK* | 59.6% | 2.1% | 77.8% | 0.0% |  | *RBL2* | 29.8% | 4.3% | 55.6% | 11.1% |
| *ITPKB* | 53.2% | 0.0% | 72.2% | 0.0% |  | *CDH8* | 29.8% | 8.5% | 55.6% | 5.6% |
| *STK4* | 48.9% | 0.0% | 72.2% | 0.0% |  | *DCAMKL1* | 27.7% | 0.0% | 44.4% | 5.6% |
| *AKT2* | 46.8% | 2.1% | 61.1% | 0.0% |  | *PHKB* | 27.7% | 2.1% | 38.9% | 11.1% |
| *MAP3K10* | 46.8% | 2.1% | 61.1% | 0.0% |  | *CSNK2A2* | 29.8% | 25.5% | 55.6% | 27.8% |
| *GRB2* | 46.8% | 2.1% | 66.7% | 0.0% |  | *BCKDK* | 27.7% | 8.5% | 22.2% | 33.3% |
| *PCTK3* | 46.8% | 0.0% | 66.7% | 0.0% |  | *FUS* | 27.7% | 8.5% | 22.2% | 33.3% |
| *RHOK* | 44.7% | 6.4% | 66.7% | 5.6% |  | *MAPK3* | 27.7% | 8.5% | 22.2% | 33.3% |
| *GAS6* | 44.7% | 6.4% | 66.7% | 5.6% |  | *PHKG2* | 27.7% | 8.5% | 22.2% | 33.3% |
| *MLLT6* | 44.7% | 4.3% | 66.7% | 0.0% |  | *TAO1* | 27.7% | 8.5% | 22.2% | 33.3% |
